# Stage anxiety and piano performance: the sequential mediating roles of self-efficacy and attentional control

**DOI:** 10.3389/fpsyg.2026.1895260

**Published:** 2026-10-09

**Authors:** Yue Sun, Zhou Zhou

**Affiliations:** Pu'er University, Pu'er, China

**Keywords:** attentional control, music performance anxiety, piano performance, self-efficacy, stage anxiety

## Abstract

**Purpose:**

This study examined the relationships among stage anxiety, self-efficacy, attentional control, and piano performance from a cognitive-affective integration perspective. Specifically, it investigated whether self-efficacy and attentional control mediated the association between stage anxiety and piano performance, both independently and sequentially.

**Methods:**

A sample of 298 piano majors from a comprehensive university and a music conservatory in Yunnan Province, China, completed standardized measures of music performance anxiety, self-efficacy, and attentional control. Piano performance was evaluated through expert ratings. Descriptive statistics, correlation analysis, hierarchical regression analysis, and bootstrap mediation analyses were conducted to examine the proposed relationships.

**Results:**

Stage anxiety was negatively associated with piano performance (*β* = −0.264, p < 0.001), self-efficacy (*β* = −0.479, *p* < 0.001), and attentional control (*β* = −0.222, *p* < 0.001). In contrast, self-efficacy was positively associated with attentional control (*β* = 0.402, *p* < 0.001) and piano performance (*β* = 0.269, *p* < 0.001). Mediation analyses revealed that self-efficacy (indirect effect = −0.129, 95% CI [−0.193, −0.071]) and attentional control (indirect effect = −0.048, 95% CI [−0.087, −0.018]) significantly mediated the relationship between stage anxiety and performance. Furthermore, a sequential mediation pathway through self-efficacy and attentional control was also significant (indirect effect = −0.042, 95% CI [−0.069, −0.020]). The total indirect effect was significant (effect = −0.219, 95% CI [−0.293, −0.152]). Neither the gender × stage anxiety interaction (*β* = −0.001, *p* = 0.976) nor the prior performance experience × self-efficacy interaction (*β* = −0.039, *p* = 0.401) was significant.

**Conclusion:**

These findings support a cognitive-affective integration framework by demonstrating that self-efficacy and attentional control serve as important psychological mechanisms linking stage anxiety to piano performance. The results extend social cognitive theory and attentional control theory to music performance contexts.

**Implications:**

The findings suggest that interventions aimed at enhancing musicians’ self-efficacy and strengthening attentional control may help improve performance outcomes under pressure. Limitations and directions for future research are also discussed.

## Introduction

Stage performance is not only an outward manifestation of musical skill but also a complex psychological process that involves the dynamic interaction of the performer’s cognitive, emotional, and physical states. In the field of piano performance, performers must execute precise motor control, musical expression, and emotional conveyance in contexts that are highly public, subject to immediate evaluation, and largely irreversible, which makes them particularly susceptible to stage anxiety or music performance anxiety (MPA) (Allen, 2013; Kinney et al., 2025). Extensive research indicates that stage anxiety has become a significant psychological factor affecting the performance quality of both music learners and professional performers; it not only undermines performance stability but also impairs musical expression, motor coordination, and attentional resource allocation, consequently reducing overall performance quality (Fernholz et al., 2019; Kenny, 2011). In recent years, with advancements in music psychology and performance science, researchers have increasingly recognized that performance errors do not arise solely from technical limitations but are also closely linked to the performer’s capacity to regulate psychological states under pressure (Zhang and Jenatabadi, 2024; Osborne et al., 2014).

Stage anxiety is an established construct in evaluative settings (Barlow, 2000), and its general features do not need to be rehearsed again here. What matters for the present argument is that in musical performance it exhibits greater situational sensitivity and domain specificity than it does in most other performance settings. Kenny (2011) notes that performance anxiety is a complex response occurring in musical or singing contexts, involving interactions among cognitive evaluation, physiological arousal, and behavioral inhibition. For pianists, given the high demands for precision, continuity, and bilateral coordination, once attentional resources are disrupted by anxiety, performers are more likely to experience rhythmic disruptions, memory lapses, motor stiffness, and restricted musical expression.

Existing research suggests that stage anxiety substantially affects the quality of musical performance. Yoshie et al. (2009) found that elevated anxiety levels alter muscle tension and motor control patterns, which in turn reduces motor precision and performance fluency. Racine et al. (2026) further noted that heightened anxiety increases error monitoring tendencies, leading performers to overfocus on technical mistakes and external evaluations, thereby creating a self-reinforcing negative cycle. At the same time, stage anxiety impairs working memory efficiency, causing limited cognitive resources to be occupied by threatening information rather than allocated to musical processing and emotional expression (Eysenck et al., 2007). In piano performance, a task that requires extremely high levels of momentary attentional control, this competition for cognitive resources is particularly pronounced.

Among the theoretical frameworks explaining how anxiety affects performance, Attentional Control Theory (ACT) provides strong explanatory power (Eysenck et al., 2007). This theory posits that anxiety reduces the efficiency of the goal-directed attentional system and increases the sensitivity of the stimulus-driven system to threatening information, thereby leading to an imbalance in attentional resource allocation (Eysenck et al., 2007). In other words, individuals in an anxious state are more easily distracted by expectations of failure, self-doubt, and external evaluative cues, which makes it difficult to maintain focus on the task. During piano performance, this shift may manifest as a transition from musical expression to error monitoring, and from motor automation to explicit motor control, ultimately disrupting the automated execution of well-learned skills. Beilock and Carr (2001) noted that excessive self-focus in high-pressure situations can lead to the failure of automated skills, a phenomenon often referred to as choking under pressure.

But not all performers show a decrease in performance when anxious. Other research indicates that psychological resources and cognitive regulation skills are important factors that can provide protection against the detrimental impact of anxiety, and self-efficacy is regarded as one of these factors. Bandura (1997) theorized that self-efficacy is the basis of people’s beliefs about their own performance capabilities, which affects their decisions, emotions, level of effort and handling stress. In musical performance settings, increased self-efficacy leads to increased feelings of control over difficult situations, lower thoughts of failure, and greater task-centeredness (Ritchie and Williamon, 2011). McCormick and McPherson (2003) also showed that self-efficacy is a stronger predictor of musical performance than was actual time spent practising.

For pianists, self-efficacy is not only related to technical confidence but also to emotional stability and cognitive control during performance. Bandura (1988) suggests a significant negative relationship between self-efficacy and anxiety; individuals with low self-efficacy are more likely to interpret performance situations as threatening, leading to avoidance and heightened tension. In contrast, high self-efficacy enhances trust in personal resources and reduces anxiety-related cognitive interference. Empirical research in music psychology indicates that students with higher performance self-efficacy are better able to maintain stable attention and positive emotional states during public performances, and they tend to outperform those with lower self-efficacy (Jiang, 2024; Zelenak, 2015).

In addition to self-efficacy, attentional control is another critical variable in explaining the mechanisms underlying stage anxiety. Attentional control refers to the ability to regulate attentional allocation, inhibit irrelevant stimuli, and maintain focus on task-relevant information (Derryberry and Reed, 2002). In high-pressure performance contexts, performers must continuously suppress distracting inputs such as external evaluations, self-doubt, and anticipation of errors; therefore, attentional control directly influences performance stability and quality. Research indicates that stronger attentional control can mitigate the negative impact of anxiety on cognitive efficiency and enhance performance stability (Eysenck et al., 2007). In musical performance, attentional control affects not only motor coordination but also the real-time processing of musical structure, rhythm, and expressive elements.

It is worth noting that there may be a theoretical linkage between self-efficacy and attentional control. According to social cognitive theory, self-efficacy enhances self-regulatory capacity in stressful situations, improving goal persistence and cognitive control (Bandura, 1997). In other words, when performers believe in their ability to perform successfully, they are more likely to maintain task-oriented attention and resist interference from threatening information. Therefore, in the process through which stage anxiety affects piano performance, self-efficacy may exert its influence by shaping attentional control. This suggests that stage anxiety not only directly impairs performance but may also indirectly reduce performance by lowering self-efficacy and subsequently weakening attentional control.

Previous research has focused on stage anxiety, self-efficacy and attentional control singularly, but there are a number of areas that still need to be explored. First, previous studies have mostly investigated direct effects and there is a lack of research on integrated pathways with multiple psychological mechanisms. Secondly, research examining the connection between self-efficacy and attentional control has mainly been done within the educational or sport psychology literature, and to a lesser extent, within piano performance settings. Thirdly, studies of performance anxiety in musical contexts tend to be based on relatively simple mediation models, and the ongoing psychological process linking stage anxiety, self-efficacy, attentional control and musical performance is not fully understood. Fourth, the conditions under which these mechanisms operate have received little attention. Prior performance experience, perceived social support, and habitual coping strategies could all plausibly qualify the strength of the anxiety-performance link (Fernholz et al., 2019; Zhang and Jenatabadi, 2024), yet they are rarely modeled in piano performance research.

Studying this mechanism in piano performance rather than in a generic “musical performance” context is theoretically consequential. The piano imposes simultaneous demands on fine motor precision, bimanual coordination, sustained memory retrieval, and expressive timing within a single, uninterrupted and largely irreversible performance act (Yoshie et al., 2009). Ensemble performers can partly externalize monitoring and timing to co-performers, and performers of many other instruments can pause, breathe, or re-enter; a solo pianist who loses attentional control cannot do any of these without an audible failure. The demands of the task therefore make the attentional consequences of anxiety unusually observable, and they allow performance quality to be indexed far more precisely than is usually possible in other performance domains. In this sense, the piano is not merely a convenient sample but a high-resolution test case for attentional control theory.

The present study follows this and investigates the effect of stage anxiety on the piano performance of pianists and also re-tests the chained mediating role of self-efficacy and attentional control. The purpose of this study is to explain the mechanisms on how stage anxiety affects performance from a cognitive-emotional integration point of view, in order to shed light on the theoretical basis for psychological interventions in music performance. This study makes three contributions. Firstly, it provides a more systematic explanation of the psychological pathways by which anxiety can influence piano performance, as the authors incorporate social cognitive theory and attentional control theory, and add a chained mediation model. Second, it considers piano playing in terms of its performance as a complex art from the perspective of music psychology research on the mechanisms of performance anxiety. Third, the study specifies the conditions under which the sequential pathway operates, by testing whether gender and prior performance experience qualify the effects and by treating the resulting boundary conditions as part of the theoretical claim rather than as a caveat.

Drawing on existing theoretical and empirical research, this study proposes the following hypotheses: First, stage anxiety will significantly and negatively predict piano performance; second, self-efficacy mediates the relationship between stage anxiety and piano performance; third, attentional control mediates the relationship between stage anxiety and piano performance; fourth, self-efficacy and attentional control jointly exert a chained mediation effect in the relationship between stage anxiety and piano performance (see Figure 1). Fifth, on an exploratory basis, gender and prior performance experience were examined as potential moderators of these pathways.

**Figure 1:**
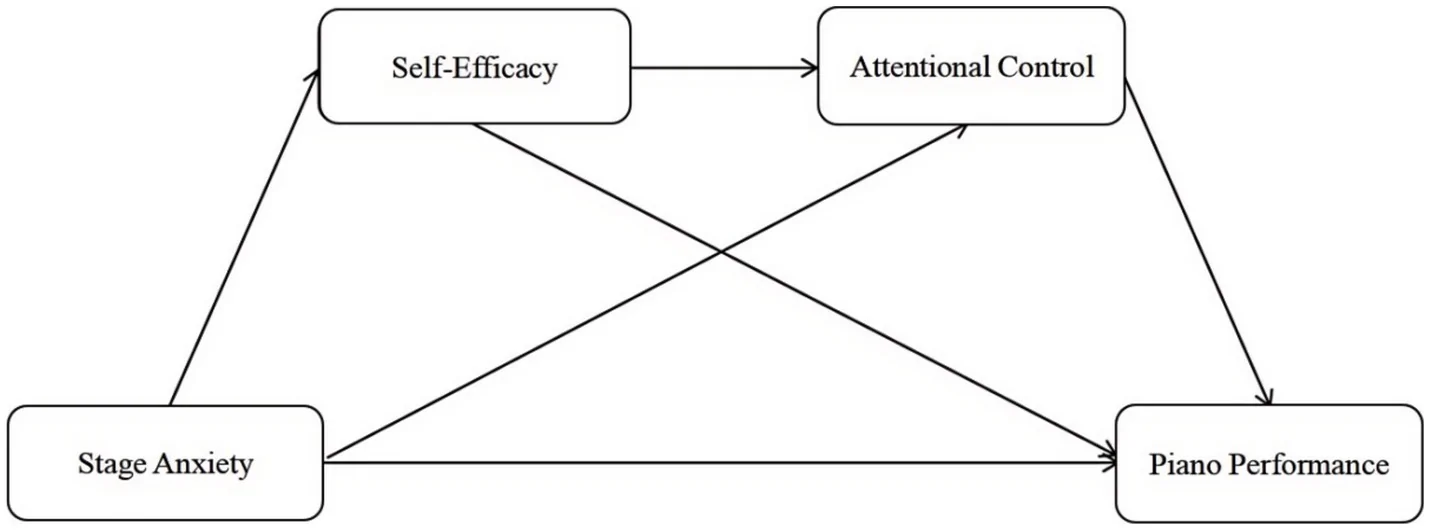
Schematic diagram of the hypothetical model.

## Research methods

### Study participants

This study used a combination of convenience and cluster sampling to recruit piano majors from Pu’er University (Yunnan Province, China) and a dedicated music conservatory in the same province. To ensure professional representativeness and sufficient stage performance experience, participants were required to meet the following criteria: (1) at least 5 years of systematic piano training; (2) experience in public performances or formal recitals within the past year; (3) good physical and mental health, with no diagnosed severe anxiety or neurological disorders; and (4) provision of informed consent and voluntary participation in the study. The health criterion in (3) was operationalized through self-report rather than through a standardized clinical assessment: participants indicated whether they were currently receiving treatment for, or had been diagnosed with, a psychiatric or neurological condition, and those who reported such a diagnosis or treatment were excluded from the analyses. No structured clinical interview or standardized symptom inventory was administered, which limits the certainty with which clinically significant anxiety can be ruled out and is returned to in the Discussion. The required sample size was determined *a priori* using G*Power 3.1 (Faul et al., 2009). Detecting a medium effect (f^2^ = 0.15) in a regression model with four predictors at *α* = 0.05 with power = 0.80 requires 85 cases, and simulation evidence indicates that stable bias-corrected bootstrap confidence intervals for medium-sized indirect effects require at least 150 cases (Fritz and MacKinnon, 2007). A target of approximately 300 cases was therefore set before data collection so that the sequential mediation model and the planned moderation tests would be adequately powered; a post-hoc check based on the observed effect size confirmed that power for the focal set of predictors in the retained sample exceeded 0.99. A total of 326 questionnaires were initially distributed. After excluding questionnaires with substantial missing data, duplicate responses, and other invalid entries, 298 valid responses were retained, yielding a response rate of 91.41%. Among the participants, 118 were male and 180 were female. The age ranged from 18 to 26 years, with a mean age of 21.61 years (SD = 2.08). The sample included 224 undergraduate and 74 graduate students.

All participants were judged to have an advanced level of piano performance based on their performance level and academic background. Most had been involved in competitions, concerts or university and/or higher level academic recitals suggesting a relatively stable range of experience in the performance of stage repertoire. Because stage anxiety and performance could both be related to the performer’s background, four background variables were recorded for subsequent statistical control: years of systematic piano training, age, gender, and level of study (undergraduate vs. postgraduate). Years of piano training averaged 8.62 years (SD = 2.50, range 5–16) and age averaged 21.61 years (SD = 2.08, range 18–26); 39.6% of the sample were male and 24.8% were postgraduate students.

### Ethical considerations

This study was conducted in accordance with the ethical principles outlined in the Declaration of Helsinki and was approved by the Ethics Committee of Pu′er University (Approval No. 2025–10). Before participation, all participants were informed about the purpose, procedures, and potential risks of the study. Written informed consent was obtained from all participants prior to data collection. Participation was voluntary, and participants were informed that they could withdraw from the study at any time without penalty. All collected data were anonymized and used exclusively for research purposes.

### Data collection procedure

Data collection was conducted after obtaining ethical approval from the institutional ethics committee of the participating university. Before participation, all participants were informed about the purpose of the study, the procedures involved, and their rights as research participants. Written informed consent was obtained from all participants before data collection.

The data collection process consisted of two stages. First, participants completed self-report questionnaires measuring stage anxiety, music performance self-efficacy, and attentional control in a quiet classroom environment. Participants were instructed to respond based on their general experiences related to piano performance situations.

Second, participants completed the piano performance task under standardized performance conditions. Each participant performed the assigned piano piece individually, and the performance was recorded for subsequent expert evaluation. The recording procedures were standardized to ensure comparable conditions across participants. After data collection, questionnaire responses and performance recordings were coded anonymously and used exclusively for research purposes.

### Research instruments

#### Anxiety scale (MPAI-A)

This study used the Music Performance Anxiety Inventory for Adolescents (MPAI-A), developed by Osborne and Kenny (2005), to assess stage anxiety among piano performers. This instrument is one of the most widely used tools in music psychology and is capable of assessing cognitive, emotional, and physiological responses in performance contexts. The scale includes dimensions such as worry, physiological tension, negative expectations, self-doubt, and avoidance behaviors.

Given the characteristics of the participants, the scale was slightly adapted to the study context to better reflect piano performance situations. Specifically, the term “music performance” was replaced with “piano performance” to ensure consistency in interpretation. The scale uses a 7-point Likert format ranging from 1 (strongly disagree) to 7 (strongly agree), with higher scores indicating higher levels of stage anxiety. Because the item wording was modified for piano performance, the adapted version was validated in the present sample rather than assumed to be equivalent to the original instrument. Within the measurement model described below, the four stage-anxiety parcels loaded on a single factor at 0.89 to 0.97, and the construct showed high composite reliability (CR = 0.96) and internal consistency (Cronbach’s α = 0.906). These results support the use of the piano-adapted scale as a measure of stage anxiety in this sample.

#### Music performance self-efficacy scale

To assess performers’ beliefs about their abilities in public performance settings, the study used the Music Performance Self-Efficacy Scale developed by Ritchie and Williamon (2011). The scale assesses perceived competence in performance skills, stage adaptability, and performance stability, and is widely used in music education and psychology research.

The scale includes items reflecting confidence under pressure and expectations of performance quality. A 5-point Likert scale is used, ranging from 1 (strongly disagree) to 5 (strongly agree), with higher scores indicating stronger self-efficacy. For this study the instrument was translated into Chinese using a forward- and back-translation procedure (Brislin, 1970), with discrepancies resolved by consensus among bilingual researchers, and the Chinese version was reviewed for clarity and relevance by three experts in music education. Previous research has shown that the scale demonstrates good construct validity and applicability across contexts (Ritchie and Williamon, 2011; Zarza-Alzugaray et al., 2020). In the present sample the three item parcels loaded on the self-efficacy factor at 0.87 to 0.96 (CR = 0.95), and Cronbach’s α was 0.889.

#### Attentional control scale (ACS)

This study used the Attentional Control Scale (ACS) developed by Derryberry and Reed (2002) to assess attentional regulation under stress. The scale assesses attentional allocation, inhibition of irrelevant stimuli, and maintenance of goal-directed attention, and is widely used in anxiety and cognitive control research.

The scale includes two dimensions: focusing and shifting. Focusing refers to maintaining attention despite distractions, whereas shifting reflects flexibility in reallocating attention across tasks. During piano performance, attentional control is essential for maintaining performance stability in the presence of internal and external distractions.

The scale uses a 4-point Likert format ranging from 1 (almost never) to 4 (always). Reverse-scored items were recoded prior to analysis, and higher scores indicate stronger attentional control. The scale was translated into Chinese using the same forward- and back-translation procedure described above. Previous studies have demonstrated good reliability and validity (Clauss and Bardeen, 2020; DeVito et al., 2019; Derryberry and Reed, 2002), although subsequent psychometric work has shown that the two original subscales are less stable than the general factor (Clauss and Bardeen, 2020). Consistent with this, in the present data the first eigenvalue (4.84) was far larger than the second (1.07) and parallel analysis retained a single factor, so the total score was used; the four parcels loaded on this factor at 0.71 to 0.79 (CR = 0.83), and Cronbach’s α was 0.845.

#### Evaluation of piano performance

Piano performance was evaluated using expert ratings. Participants performed standardized piano pieces selected according to their advanced training level and performance experience. The selected repertoire was at an intermediate-to-advanced difficulty level appropriate for university-level piano students and required comprehensive musical skills, including technical accuracy, rhythmic control, tonal refinement, musical interpretation, and expressive communication. The repertoire was selected to ensure that performance evaluation reflected overall musical competence rather than isolated technical ability. Restricting the task to a single standardized piece was a deliberate design decision: holding repertoire difficulty, technical demand, and duration constant ensures that between-performer differences in rated quality can be attributed to psychological state rather than to differences in the difficulty or familiarity of contrasting repertoire. This choice favors internal validity, and its cost is acknowledged here, since a single-piece protocol samples less of the sustained attentional demand of a full recital program and therefore limits ecological validity; the point is revisited among the limitations below.

Recording equipment, acoustic environment, and lighting were controlled to minimize external variability. All recordings were anonymized prior to evaluation.

Performance was rated by five senior experts in piano performance and music education. The evaluation covered five dimensions: technical accuracy, rhythmic stability, tone control, musical expressiveness, and stage presence. Each dimension was scored on a 10-point scale, and final scores were calculated as the mean across raters. The five raters were senior experts in piano performance and music education, and they received no information about participants’ questionnaire responses.

Inter-rater reliability was assessed using the intraclass correlation coefficient, yielding a value of ICC = 0.877, indicating high consistency. To reduce subjective bias, all raters received standardized training on a common rubric prior to scoring. Two specific rater biases were also considered. First, familiarity: raters were not given participants’ names or institutional affiliations and evaluated anonymised recordings, which reduces but cannot fully eliminate familiarity effects, given that raters and participants were drawn from the same regional music community. Second, interpretive style: rater-level means were almost identical (6.055 to 6.088 on the 10-point scale, a maximum deviation of 0.02 points, or 0.2% of the scale range, from the panel grand mean of 6.073), indicating no systematic leniency or severity differences among raters. The ratings nonetheless remain one panel’s judgment of one performance occasion.

### Data analysis

Data were analyzed in SPSS 26.0 and AMOS 26.0. To analyze the relationships between variables, descriptive statistics and correlation analyses were performed. Construct validity was tested using CFA and reliability in terms of internal consistencies was tested using Cronbach’s alpha. To determine whether common method bias existed, Harman’s one-factor test was performed for all the data. Global model fit was evaluated using the comparative fit index (CFI), the Tucker–Lewis index (TLI), the root mean square error of approximation (RMSEA), and the standardized root mean square residual (SRMR); CFI and TLI values above 0.90, RMSEA below 0.08, and SRMR below 0.08 were treated as acceptable (Hu and Bentler, 1999).

A path analysis model was used to test the proposed sequential mediation model. Stage anxiety was designated predictor (X), piano performance as outcome (Y), self-efficacy as mediator (M1) and attentional control as mediator (M2). Measures were freely estimated for errors of measurement. To evaluate the global fit of this model, it was also estimated as a latent-variable structural equation model in which each construct was represented by item parcels (four parcels for stage anxiety, three for self-efficacy, and four for attentional control) and piano performance by the five rated dimensions. Parcels were used both to keep the number of estimated parameters proportionate to the sample size and because preliminary item-level diagnostics indicated local dependence among consecutively administered items within each scale.

The bootstrap method with 5,000 resamples was employed to obtain parameter estimates and confidence intervals in case of non-normality. The mediating effect was deemed to be significant when the 95% confidence interval was not equal to zero. Standardized coefficients (*β*), R^2^, and 95% confidence intervals are reported throughout, and the magnitude of the correlations is interpreted using Cohen’s (1988) benchmarks.

Background variables were included as covariates in the regression models to ensure the robustness of the findings: gender, age, years of piano training, and level of study were entered as a first block, with the focal predictors added as a second block. Finally, three interaction terms were tested to examine whether the paths were conditional on individual differences: gender × stage anxiety, prior performance experience × self-efficacy, and prior performance experience × stage anxiety. All predictors were standardized before the products were computed, the interaction terms were entered after the main effects, and these tests were exploratory, so null results are reported rather than omitted.

## Results

### Descriptive statistics and correlation analysis

Descriptive statistics and Pearson correlation analyses were conducted for all core variables, and the results are presented in Table 1. Stage anxiety (*M* = 4.08, SD = 1.23) was at a moderate-to-high level, whereas self-efficacy (*M* = 2.96, SD = 0.83) and attentional control (*M* = 2.50, SD = 0.52) were relatively low. The mean score for piano performance was 6.07 (SD = 1.72).

**Table 1:** Descriptive statistics and correlation analysis.

Variable	*N*	*M*	SD	1	2	3
1. Stage anxiety	298	4.08	1.23	1		
2. Self-efficacy	298	2.96	0.83	−0.48**	1	
3. Attentional control	298	2.50	0.52	−0.42**	0.51**	1
4. Piano performance	298	6.07	1.72	−0.48**	0.51**	0.46**

**p < 0.01.

Correlation analysis indicated that the associations among variables were consistent with the study hypotheses. Specifically, stage anxiety was significantly negatively correlated with piano performance (*r* = −0.48, *p* < 0.01), suggesting that higher stage anxiety was associated with lower performance. Stage anxiety was also negatively correlated with both self-efficacy (*r* = −0.48, *p* < 0.01) and attentional control (*r* = −0.42, *p* < 0.01), indicating that higher anxiety was associated with lower self-efficacy and weaker attentional control.

In contrast, self-efficacy was positively correlated with attentional control (*r* = 0.51, *p* < 0.01) and piano performance (*r* = 0.51, *p* < 0.01). Attentional control was also positively correlated with piano performance (*r* = 0.46, *p* < 0.01). These results provide preliminary support for subsequent analyses of the proposed sequential mediation model involving self-efficacy and attentional control. Interpreted against Cohen’s (1988) benchmarks, the anxiety-performance association (*r* = −0.48) and the self-efficacy-performance association (*r* = 0.51) are moderate to strong, the self-efficacy-attentional control association (*r* = 0.51) is strong, and the attentional control-performance association (*r* = 0.46) is moderate. The anxiety-performance correlation alone accounts for approximately 23% of the variance in rated performance, which is a substantial share for a single psychological predictor of an externally rated artistic outcome.

### Chain mediation model

Hierarchical regression analysis was used to examine the mediating roles of self-efficacy and attentional control in the association between stage anxiety and piano performance (see Table 2).

**Table 2:** Results of hierarchical regression analysis.

Dependent variable	Predictor	*β*	SE	*t*	R^2^
Self-efficacy (M1)	Stage anxiety (X)	−0.479	0.051	−9.339***	0.235
Attentional control (M2)	Stage anxiety (X)	−0.222	0.056	−3.971***	0.303
	Self-efficacy (M1)	0.402	0.056	7.181***	
Piano performance (Y)	Stage anxiety (X)	−0.264	0.054	−4.841***	0.375
	Self-efficacy (M1)	0.269	0.058	4.666***	
	Attentional control (M2)	0.217	0.056	3.904***	

***p < 0.001.

First, analysis showed that stage anxiety was significantly negatively associated with self-efficacy (*β* = −0.479, *p* < 0.001), explaining 23.5% of the variance. Second, when attentional control was treated as the dependent variable, stage anxiety and self-efficacy were entered into the model. Stage anxiety was negatively associated with attentional control (*β* = −0.222, *p* < 0.001), whereas self-efficacy was positively associated with attentional control (*β* = 0.402, *p* < 0.001), with an R^2^ of 0.303.

Finally, in the full model including the mediators, stage anxiety, self-efficacy, and attentional control were all significantly associated with piano performance (*p* < 0.001), and the model explained 37.5% of the variance. Notably, after the inclusion of mediating variables, the standardized coefficient of stage anxiety decreased from −0.482 to −0.264, while remaining statistically significant.

This pattern suggests that self-efficacy and attentional control function as mediators, and that the mediation is partial. In addition, the X × M interaction terms were non-significant (*p* > 0.05), which is consistent with the specification of parallel and sequential mediation paths (see Figure 2). The hypothesized structural model fitted the data well, χ^2^(98) = 207.46, CFI = 0.984, TLI = 0.981, RMSEA = 0.061, SRMR = 0.033; standardized parcel loadings ranged from 0.71 to 0.99, and all six structural paths were significant and in the expected direction (standardized estimates from |0.23| to |0.48|).

**Figure 2:**
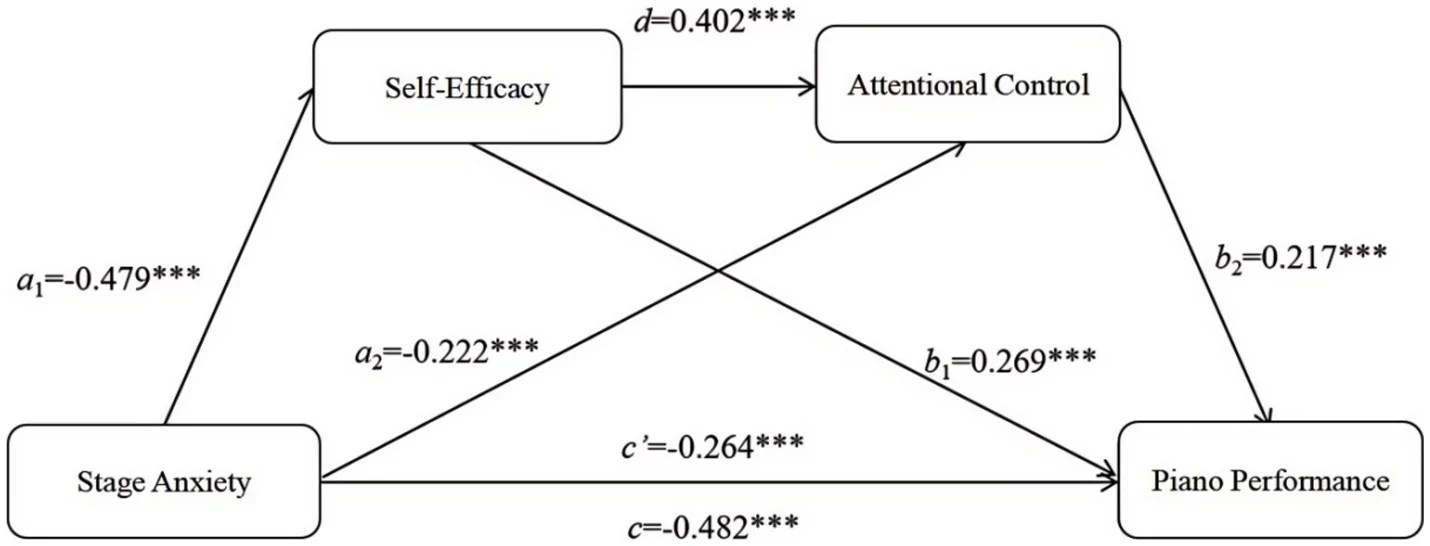
Verification model diagram. ***p < 0.001.

With respect to the covariates, the four background variables explained only 1.3% of the variance in rated performance when entered on their own, *F*(4, 293) = 0.95, *p* = 0.435, and none of them was significantly associated with performance (gender: *β* = −0.024, *t* = −0.42, *p* = 0.675; age: *β* = −0.092, *t* = −1.58, *p* = 0.116; years of piano training: *β* = 0.021, *t* = 0.36, *p* = 0.719; level of study: *β* = −0.062, *t* = −1.07, *p* = 0.286). After the focal predictors entered the model the covariates remained non-significant (gender: *β* = 0.006, SE = 0.047, *t* = 0.12, *p* = 0.904; age: *β* = −0.079, SE = 0.047, *t* = −1.68, *p* = 0.094; years of piano training: *β* = 0.027, SE = 0.047, *t* = 0.58, *p* = 0.563; level of study: *β* = −0.033, SE = 0.047, *t* = −0.71, *p* = 0.482), while the three focal predictors added 36.2% of incremental variance, ΔF(3, 290) = 55.89, *p* < 0.001. The standardized total effect of stage anxiety on performance was essentially unchanged when the covariates were included (*β* = −0.483 without covariates vs. *β* = −0.482 with them), indicating that the relationships reported here are not attributable to differences in gender, age, training, or level of study.

Three exploratory interaction tests examined whether the paths were conditional on individual differences. None reached significance: the gender × stage anxiety term predicting piano performance was non-significant (*β* = −0.001, SE = 0.048, *t* = −0.03, *p* = 0.976), as was the prior performance experience × self-efficacy term (*β* = −0.039, SE = 0.047, *t* = −0.84, *p* = 0.401) and the prior performance experience × stage anxiety term (*β* = −0.025, SE = 0.047, *t* = −0.54, *p* = 0.587). The absence of moderation indicates that the sequential pathway operated similarly for men and women and across levels of prior performance experience within this sample. It should not be read as evidence of invariance, because the study was powered for main effects rather than for interactions and only three of the plausible moderators were tested.

### Bootstrap mediation analysis

A bias-corrected bootstrap method with 5,000 resamples was used to further examine the robustness of the mediation effects (see Table 3). The results indicated that the total indirect effect was −0.219, with a 95% confidence interval of [−0.293, −0.152]. As the interval did not include zero, this suggests that the total indirect effect via self-efficacy and attentional control was statistically significant.

**Table 3:** Bootstrap results for mediation effects.

Effect type	Path	Effect	SE	95% CI lower	95% CI upper
Direct effect	X → Y	−0.264	0.054	−0.371	−0.156
Total indirect effect	—	−0.219	0.036	−0.293	−0.152
Indirect effect 1	X → M1 → Y	−0.129	0.032	−0.193	−0.071
Indirect effect 2	X → M2 → Y	−0.048	0.018	−0.087	−0.018
Indirect effect 3	X → M1 → M2 → Y	−0.042	0.013	−0.069	−0.020

X, stage anxiety; M1, self-efficacy; M2, attentional control; Y, piano performance. Confidence intervals that do not include zero indicate statistically significant effects.

In the analysis of specific indirect paths, all three indirect effects were significant. The indirect effect for the path X → M1 → Y was −0.129, with a 95% confidence interval of [−0.193, −0.071]. The indirect effect for X → M2 → Y was −0.048, with a 95% confidence interval of [−0.087, −0.018]. The sequential mediation path X → M1 → M2 → Y yielded an indirect effect of −0.042, with a 95% confidence interval of [−0.069, −0.020].

## Discussion

This study explored, from a cognitive-affective integration perspective, the psychological mechanisms linking stage anxiety and piano performance and examined the sequential mediating roles of self-efficacy and attentional control. Results confirmed a significant negative association between stage anxiety and performance and provided evidence for a sequential pathway in piano performance: stage anxiety → self-efficacy → attentional control → performance. These findings offer a theoretical perspective on the phenomenon of “choking” in musical performance and inform potential intervention strategies. The moderation tests that were conducted were null, which suggests that within this sample the pathway is not conditional on gender or on prior performance experience and is better described as a general rather than a subgroup-specific process.

Firstly, the study confirms that stage anxiety is an important factor affecting piano performance. This is consistent with Kenny (2011) and Fernholz et al. (2019), who found there is a multi-faceted nature of anxiety as a negative emotional state, which can affect performance in a number of ways. It causes physiological tension, muscle stiffness (Yoshie et al., 2009), and decreases the cognitive expressiveness. When self-efficacy and attentional control were taken into account, direct influence of stage anxiety on performance was still significant, suggesting that stage anxiety might influence performance through other unmeasured variables, such as physiological arousal or motivational inhibition.

Second, the dual and sequential mediation of self-efficacy and attentional control is emphasized. The present study identified three important indirect pathways: two simple mediation pathways (self-efficacy and attentional control) and one sequential mediation pathway (self-efficacy → attentional control). The route through self-efficacy was the largest effect size, highlighting the importance of this as a fundamental resource for protection. These findings indicate that Bandura's (1988, 1997) social cognitive theory holds true: performers with higher self-efficacy are less sensitive to negative expectations and excessive self-monitoring (Racine et al., 2026), and can thus concentrate more on the musical task itself. The results are also consistent with those of Jiang (2024) and Zhang and Jenatabadi (2024), which highlight the significance of self-efficacy in music learning and performance.

Furthermore, this study is among the first in piano performance research to examine the relationship between self-efficacy and attentional control and to construct a comprehensive sequential mediation model. Stage anxiety not only directly impairs attentional control but also indirectly affects it through reduced self-efficacy. Integrating social cognitive theory with Attention Control Theory (ACT) (Eysenck et al., 2007), the results indicate that high self-efficacy enables performers to allocate cognitive resources effectively and suppress anxiety-related distractions, maintaining focus on the performance. Conversely, low self-efficacy amplifies the negative effects of anxiety on attention, creating a cycle of anxiety → low self-efficacy → attentional distraction → performance decline. This chain illustrates how macro-level emotional beliefs influence micro-level cognitive control, ultimately affecting behavior, thus extending our understanding of psychological mechanisms in musical performance.

The evidence base for these conclusions is, however, more fractured than the preceding discussion implies, and three points of tension deserve explicit acknowledgement. First, the anxiety-performance relationship is not always negative. Inverted-U and catastrophe models predict that moderate arousal can facilitate performance (Hardy and Parfitt, 1991; Woodman and Hardy, 2003), and musicians themselves often report that anxiety becomes facilitative once it is directed at the task. The present data cannot distinguish debilitative from facilitative anxiety, because the MPAI-A yields only a total intensity score; the negative coefficient reported here may therefore describe average intensity rather than a uniformly harmful process. Second, the mediating role of self-efficacy is not universally supported. In other achievement domains the unique contribution of self-efficacy to performance frequently shrinks once prior achievement or practice history is partialled out, and its effect can be conditional on contextual resources such as perceived social support (Zarza-Alzugaray et al., 2020; Zhang and Jenatabadi, 2024). The present study cannot rule out a similar attenuation, although the covariate analyses reported above suggest that practice-related variables did not account for the association in this sample. Third, the causal ordering assumed here—anxiety → self-efficacy → attentional control → performance—is one of several defensible orderings; a plausible alternative is that relatively stable attentional capacity shapes the appraisal that produces anxiety in the first place. A cross-sectional design cannot adjudicate between these possibilities, and the fit indices reported above, while acceptable, do not by themselves rule out equivalent models. The contribution of the present study is therefore best understood as the identification of a plausible and well-fitting mechanism rather than as a demonstration of its causal priority.

The implications of this research for music education and music performance psychology are practical, but they should be stated with appropriate caution because no intervention was manipulated in this study. Although many traditional strategies focus on reducing anxiety (e.g., relaxation training), a more basic strategy may be to increase performers’ self-efficacy. To build confidence, mastery experiences, vicarious learning, verbal persuasion, and emotional control (Bandura, 1997) can be used by educators. Attentional control, whether in the form of mindfulness or cognitive flexibility, should be part of a performance psychologist’s training to help improve the focus in a stressful situation. Top-down (belief enhancement) and bottom-up (cognitive control) may be a more effective way to help pianists reduce stage anxiety and reach their peak performance. Translated into concrete procedures, the sequential model implies a specific order of operations. Self-efficacy is most reliably built through mastery experience, so graded exposure—beginning with low-stakes studio performances and progressively increasing audience size and evaluative salience—is a defensible first step, supplemented by video-based self-modeling and process-focused rather than outcome-focused feedback. Reinterpretation of arousal (reading it as readiness rather than threat) is a further low-cost technique that targets the appraisal stage of the model. Attentional control training should then follow, since the model suggests that attentional resources become trainable only once performers no longer appraise the situation as threatening: brief mindfulness practice, pre-performance attentional routines, and simulated-distraction rehearsal all load on the focusing and shifting components measured here. Two caveats limit the strength of these recommendations. First, evidence that these techniques transfer to objectively rated performance is much weaker than the evidence that they reduce self-reported anxiety; systematic reviews of interventions for music performance anxiety report consistent improvements in anxiety but smaller and less consistent gains in performance quality (Kinney et al., 2025). Second, the effect sizes reported here imply that a one-standard-deviation reduction in stage anxiety would be associated with roughly half a standard deviation of improvement in rated performance, so such training should be presented to students as one component of a broader preparation strategy rather than as a remedy.

This study also has several limitations. Future studies may include longitudinal or experimental design as the cross section designs are not causal. Even though expert ratings were used, there may be some subjective bias that could be incorporated into future studies by utilizing objective measures of performance (such as MIDI data). Lastly, because the sample population were primarily piano majors at the university, care must be taken in extrapolating to professional musicians or other musicians performing on instruments other than piano. In addition, because the health criterion was established by self-report rather than by a standardized clinical assessment, participants with undiagnosed or unreported clinical anxiety cannot be ruled out. The study was also not powered to detect moderation, and other plausible moderators—perceived social support, coping style, and personality—were not measured.

A further limitation is cultural. All participants were recruited in Yunnan Province, China, and all measures were administered in Chinese. The models that frame this study were developed largely in Western contexts, and the meaning of a public performance, the acceptability of displaying anxiety, and the availability of mental-skills training all differ across educational cultures (Kenny, 2011). In Chinese music education, where advancement is closely tied to high-stakes auditions and examinations, the social-evaluative component of performance anxiety may be weighted more heavily than in the settings from which the original instruments were derived. Because the adapted scales were validated only in this sample, measurement invariance across cultures was not tested, and the magnitude of the paths reported here should be treated as locally estimated rather than universally generalisable until cross-cultural replication is available.

In general, this study demonstrates the complicated psychological processes involved in the influence of stage anxiety on piano performance. It expands on previous studies on a single mediator examined and offers a starting point to building a full-fledged model of music performance psychology. Future research could explore other possible moderators (e.g., personality or social support), or use neuroscientific assessment techniques (e.g., EEG or fNIRS) to assess cognitive processes during performance, to better inform a multi-level understanding of how stage anxiety is related to artistic performance.

## Conclusion

This study demonstrates that stage anxiety is negatively associated with piano performance and that this relationship is mediated by self-efficacy and attentional control, both independently and sequentially. The findings highlight the importance of cognitive and attentional processes in understanding performance under pressure and suggest potential directions for targeted interventions in music education.

## Data Availability

The raw data supporting the conclusions of this article will be made available by the authors, without undue reservation.
